# Cancer Associated Fibroblasts Promote Renal Cancer Progression Through a TDO/Kyn/AhR Dependent Signaling Pathway

**DOI:** 10.3389/fonc.2021.628821

**Published:** 2021-03-25

**Authors:** Li-bo Chen, Shun-ping Zhu, Tian-pei Liu, Heng Zhao, Ping-feng Chen, You-jun Duan, Rong Hu

**Affiliations:** ^1^ Department of Urology, The First Affiliated Hospital of University of South China, Hengyang, China; ^2^ Department of Respiratory, The First Affiliated Hospital of University of South China, Hengyang, China; ^3^ Department of Radiology, The First Affiliated Hospital of University of South China, Hengyang, China

**Keywords:** cancer associated fibroblasts, kynurenine, aromatic hydrocarbon receptor, renal cancer, tryptophan 2, 3-dioxygenase (TDO)

## Abstract

Cancer associated fibroblasts (CAFs) play crucial roles in cancer development, however, the specific mechanisms of CAFs associated renal cancer progression remain poorly understood. Our study observed enriched CAFs in high degree malignant tumor tissues from renal cancer patients. These CAFs isolated from tumor tissues are prone to facilitate drugs resistance and promote tumor progression *in vitro* and *in vivo*. Mechanistically, CAFs up-regulated tryptophan 2, 3-dioxygenase (TDO) expression, resulting in enhanced secretion of kynurenine (Kyn). Kyn produced from CAFs could up-regulated the expression of aromatic hydrocarbon receptor (AhR), eventually resulting in the AKT and STAT3 signaling pathways activation. Inhibition of AKT signal prevented cancer cells proliferation, while inhibition of the STAT3 signal reverted drugs resistance and cancer migration induced by kynurenine. Application of AhR inhibitor DMF could efficiently suppress distant metastasis of renal cancer cells, and improve anticancer effects of sorafenib (Sor)/sunitinib (Sun), which described a promising therapeutic strategy for clinical renal cancer.

## Introduction

Renal cancer is one of most common urogenital neoplasms and has a high risk of distant metastasis (1). Although standard surgery plus novel tyrosine kinases inhibitors, including Sun or Sor, have reported to improve the outcome and prognosis of renal cancer patients in recent years (2). However, many patients still suffered from tumor recurrence and distant metastasis due to the drugs resistance and sustained tumor growth (3). More importantly, it remains poorly understood in the underlying mechanism of renal cancer progression.

Tumor progression is a complex set of proliferative disease, which is determined by diverse factors, including tumor heterogeneity (4), extracellular matrix (5) and tumor microenvironment (6). Several stromal or immune cells in tumor microenvironment, such as myeloid inhibitory cells (7), tumor associated macrophages (8), mesenchymal stem cells (9) and CAFs (10), have been reported to participate in the tumor progression. Among those cell subpopulations, CAFs have been observed in most solid tumor tissues. Increasing studies suggest that CAFs serves as stromal cells to support cancer development. Previous reports have provided evidence to suggest that cytokines derived from CAFs play crucial roles in regulating tumor behaviors. For example, CAFs are capable of promoting tumor stemness and facilitating cancer cells proliferation through secretion of IGF2 (11). Meanwhile, CAFs could mediate angiogenesis and tumor microenvironment remodeling though secretion of VEGF (12). Clinical and pathological studies also suggested that the distribution of CAFs is tightly correlated to the pro-survival signaling pathways activation in tumor cells (13). However, the potential role of CAFs in renal cancer remains poorly understood and the specific underlying mechanisms of CAFs in renal cancer development have yet to be explored.

In our study, we observed increasing number of CAFs in those high degree malignant tumor tissues from renal cancer patients. Those CAFs isolated from tumor tissues were capable of promoting renal cancer cells proliferation and migration. Meanwhile, we proved that those CAFs also mediated the drugs resistance of renal cancer cells, resulting in poor prognosis in renal cancer patients. We further disclosed the underlying mechanism of CAFs induced tumor progression, which is dependent on a TDO/Kyn/AhR pathway. Our study described that blockade of AhR signals could efficiently improve the outcome of traditional Sor/Sun therapy, which provided a novel sight in clinical renal cancer therapy.

## Materials and Methods

### Cell Lines and Reagents

Murine renal cancer cells Renca, human renal cancer cells A498, human skin fibroblasts HFL1 and murine NIH-3T3 fibroblasts were purchased from Cell Bank of Chinese Academy of Sciences (Shanghai, China). All fibroblasts were cultured in DMEM complete culture medium (Gibico, MA, USA) supplemented with 10% fetal bovine serum (Gibco, MA, USA). A498 cells were cultured in 1640/RMPI complete culture medium (Gibico, MA, USA) supplemented with 10% fetal bovine serum (Gibco, MA, USA). Renca cells were cultured in IMDM culture medium (Gibico, MA, USA) supplemented with 10% fetal bovine serum (Gibco, MA, USA). Kyn and Kyn concentration detection Kit were purchased from Abcam (Cambridge, UK). Sorafenib and sunitinib were purchased from Sangon (Shanghai, China). TDO inhibitor LM10, AhR inhibitor PDM2 and DMF, AKT inhibitor capivasertib (Cap) and STAT3 inhibitor S1-109 were purchased from MedChemExpress (L.A, USA).

### Patients’ Tumor Tissues and CAFs Collection

Renal tumor samples were obtained after the surgery sterilely at the First Affiliated Hospital, University of South China. All samples were reviewed by a pathologist according to World Health Organization classification. Samples were divided into non-metastatic and metastatic groups according to the subsequent clinical follow-up. Samples were divided into high degree (H-D, stage I and II) and low degree (L-D, stage III and IV) groups according to the pathological diagnose. All samples collection and following analysis were performed according to the declaration of Helsinki and Ethical approval was obtained from the committee of the First Affiliated Hospital, University of South China. For CAFs isolation, tumor tissues from patients or Renca-bearing BalB/C mice were collected and cut into pieces as small as possible. Those tissues were further digested by ACCUMAXTM (Sigma, MA, USA) at 37°C, 5% CO_2_ incubator for 1~2 hours. The digested cells were collected by filtration (40 μm, Thermo, MA, USA). Half of the cell suspension was stained by CD45 (eBioscience, MA, USA) and CD90 (eBioscience, MA, USA) for cytometry analysis. The rest cells suspension was collected and seeded into 6-well plate containing 2 ml DMEM medium with 10% fetal bovine serum for overnight at 37°C. 12 hours later, the medium was replaced with fresh medium to remove the un-adherent cells. After 2~4 passages, the CD45+/CD90+ positive CAFs were sorted for α-SMA analysis and co-culture. The isolated CAFs were maintained for at most 20 passages.

### Cell Proliferation Detection

Cell proliferation of Renca and A498 were examined by MTT Kit (Solarbio, Beijing, China). Briefly, 2000 tumor cells were seeded into 96-well plate after treatment of CAFs, fibroblasts or signals inhibitors (as described in figure legends). After 0, 24, 48 and 72 hours, 10 μl of MTT solution was added into the 96 wells, following with 3 hours incubation of 2 hours at 37°C. Absorbance was measured at 560 nm on a microplate reader (Bio-Rad, MA, USA). Each experiment was performed for independent three times.

### Transwell Analysis

Cells migrating assay was performed using 8-μm transwell chambers (Thermo, MA, USA). Renca or A498 cells were pre-treated with CAFs, fibroblasts or other inhibitors. Then, 2 × 10^4^ cells in 150 ul non-serum culture medium were seeded into the upper chambers. 800 ul culture medium containing 10% FBS medium was added into the lower chambers. 24 hours later, transwells were washed with PBS, fixed with formalin and stained with 0.1% crystal violet. Cells were counted in 3 random fields per well under a 100 × microscope. Each experiment was performed in triplicate.

### Cytotoxicity Analysis

Cytotoxicity of Sor and Sun to tumor cells was analyzed using the FITC-Annexin V/PE-PI apoptosis detection kit (BD, NJ, USA). Renca cells were treated with Sun (10 μM) or Sor (5 μM) for 48 hours. A498 were treated with Sor (20 μM) or Sun (15 μM) for 48 hours. Then tumor cells were collected and resuspended with 100 μl staining buffer after chemotherapy treatment. 5 μl FITC-Annexin V staining solution and 2 μl PE-PI staining solution were added into the cells. After incubation of 15 minutes at room temperature, the cells were washed with PBS and detected by flow cytometry on a C6 flow cytometer (BD, NJ, USA). Each experiment was performed in triplicate.

### Western Blotting

The A498 cells and Renca cells were collected for protein samples with NP40 solution. 25 μg protein samples were separated by SDS-PAGE, followed with transferring to PVDF membranes. Next, samples were examined by immunoblotting with primary antibodies against: α-SMA (1:1000, Abcam, Cambridge, UK), AhR (1:5000, Abcam, Cambridge, UK), phosphorylated-Src (1:500, Abcam, Cambridge, UK), total Src (1:500, Abcam, Cambridge, UK), phosphorylated AKT (1:500, Abcam, Cambridge, UK), total AKT (1:500, Abcam, Cambridge, UK), phosphorylated STAT3 (1:500 Abcam, Cambridge, UK), total STAT3 (Abcam, Cambridge, UK) and β-actin (1:1000, Abcam, Cambridge, UK) respectively at 4°C overnight. Then HRP-conjugated secondary antibody (1:1000, Abcam, Cambridge, UK) was incubated for 1 hour at room temperature, and visualized by using ECL detection kit (Thermo, MA, USA).

### Immunofluorescent Staining

Tumor tissues from patients were kept in 4% PFA overnight, then processed, embedded in paraffin, and sectioned at 4 μm for further study. Sections of tumor tissues from patients were blocked with blocking solution containing 5% bovine serum albumin for 30 min and stained with primary antibodies AhR (1:100; Abcam, Cambridge, UK), phosphorylated AKT (1:300, Abcam, Cambridge, UK) and phosphorylated STAT3 (1:500 Abcam, Cambridge, UK) at 4°C overnight, followed with incubation with secondary antibodies (1:1000; Abcam, Cambridge, UK) for 1 hour at room temperature. Nuclei were stained with the DAPI solution (1 µg/ml). Confocal microscope (Olympus, Tokyo, Japan) was used to visualize the sections. The fluorescence intensity of section and relative protein expression was analyzed by image J software 1.8.0 (NIH, Bethesda, Maryland, USA).

### Kyn Concentration Analysis

Kyn secretion was detected using a Human Kynurenine Elisa Kit (JIANGLAI, Shanghai, China). Briefly, 10^5^ CAFs, HSF or NIH-3T3 cells were cultured in 2 ml culture medium at 37 °C, 5% CO2 incubator. After 48 hours, the culture medium was collected and analyzed for the Kyn concentration. The Kyn concentration analysis were performed according to the guidelines of Human Kynurenine Elisa Kit. Each experiment was performed in three independent times.

### Animal Experiments Protocols

6~8 weeks female BalB/C and NOD-SCID mice were purchased from Huafukang Company (Beijing, China) and maintained in the SPF level. For lung metastasis analysis, 1×10^6^ Renca cells were injected into BalB/C mice by tail vein. On day 20, mice were sacrificed for lung metastasis analysis. For tumor suppression analysis, we established Renca subcutaneous tumor model by injecting 5×10^5^ Renca cells into BalB/C mice subcutaneously. We established A498 subcutaneous tumor model by injecting 5×10^6^ A498 into NOD-SCID mice subcutaneously. Since day 10, mice treated with PBS, Sor (15 mg/kg), Sun (20 mg/kg), DMF (10 mg/kg) or combination twice a week. The overall survival of tumor bearing mice was recorded since day 25. The calculation formula of tumor volume: tumor volume = length × width ^2^/2. All our animal experiments were conducted in accordance with guidelines of Animal Ethics Committee and approved by the Institute Ethics Committee of the First Affiliated Hospital, University of South China.

### Statistical Analysis

Each experiment was performed for at least three independent times. Results were presented as the mean ± SEM and statistical significance was analyzed using GraphPad 6.0 software (La Jolla, California, USA). Statistical significance between groups was calculated by Student’s t test for two groups or by one-way ANOVA for more than two groups. Bonferroni analysis were further used for the *post hoc* test. The correlation analysis was performed using GraphPad 6.0 software (La Jolla, California, USA). The survival rates were determined by Kaplan–Meier survival analysis (*p < 0.05; **p < 0.01; ***p < 0.001; ns, no significant difference).

## Results

### CAFs Promoted Tumor Progression in Renal Cancer

**Figure 1:**
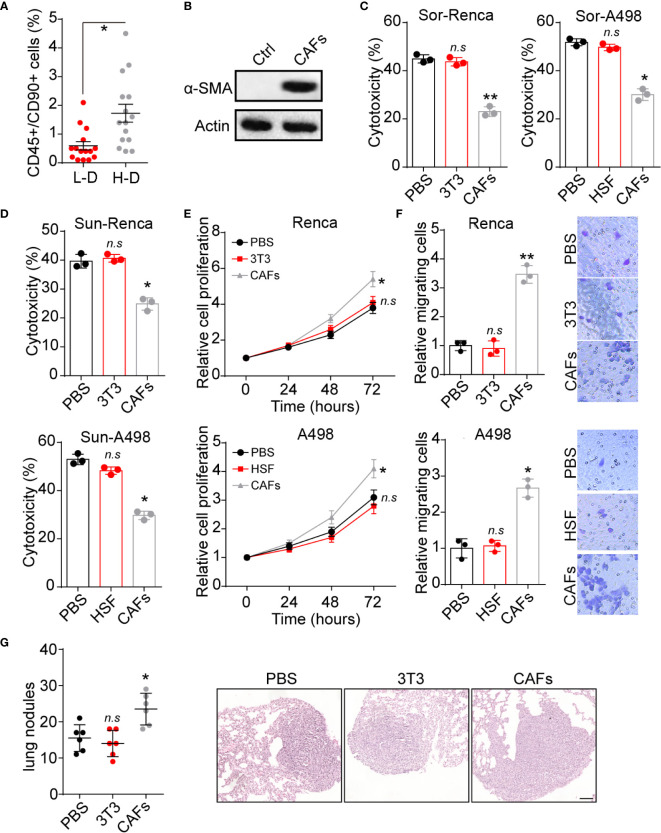
CAFs promoted renal cancer progression. **(A)** The percentage of CD45+/CD90+ cells subpopulation in tumor tissues from high degree (H-D) and low degree (L-D) malignant renal patients (n=15). **(B)** The western blotting of α-SMA in tumor tissues (ctrl) and CAFs isolated from tumor tissues of renal patients. **(C)** The cytotoxicity of Sor to Renca/A498 co-cultured with NIH-3T3, HSF or CAFs. **(D)** The cytotoxicity of Sun to Renca/A498 co-cultured with NIH-3T3, HSF or CAFs. **(E)** The relative cells proliferation of Renca/A498 co-cultured with NIH-3T3, HSF or CAFs. **(F)** The relative migrating cells numbers and representative images of Renca/A498 co-cultured with NIH-3T3, HSF or CAFs by transwell. **(G)** The lung nodules numbers and representative images of Renca lung metastasis mice models. Renca cells were co-cultured with NIH-3T3 or CAFs and 10^6^ Renca were injected into BalB/C mice by tail vein, which were sacrificed on day 20. The scale bar is 200 μm. Mean ± SEM, n.s, no significant difference; *p < 0.05, **p < 0.01.

Increasing studies have provided evidence that CAFs could regulate tumor progression through secretion of pro-survival cytokines (14). To explore the potential roles of CAFs in renal cancer development, we isolated CD45+/CD90+ cells from tumor tissues and examined their expression of α-SMA (a marker of fibroblasts), in which a higher percentage of CD45+/CD90+ cells was observed in H-D group (**Figure 1A**). Meanwhile, those CD45+/CD90+ cells revealed enhanced α-SMA expression (**Figure 1B**). Those results suggested that high degree malignant tumor tissues possessed a higher proportion of CAFs, which might be associated with the renal cancer progression. Based on this point, we seeded renal cancer cells Renca/A498 into a 24 well plate with a tranwell inert (3 μm) containing CAFs to establish tumor cells/CAFs co-culture system. Notably, tumor cells co-cultured with CAFs revealed strengthened Sor resistance, whereas no similar results were observed in normal fibroblasts cells HSF or NIH-3T3 cells groups (**Figure 1C**). Intriguingly, Renca and A498 also revealed Sun resistance after co-culture with CAFs (**Figure 1D**), indicating that CAFs could mediate the multi-drugs resistance in renal cancer. Additionally, CAFs co-culture promoted cells proliferation of Renca and A498 cells *in vitro* (**Figure 1E**). Next, we further examined the role of CAFs in renal cancer metastasis. The transwell analysis implicated that Renca and A498 cells possessed enhanced capability of migration after CAFs co-culture (**Figure 1F**). Consistently, Renca cells treated with CAFs revealed elevated lung metastasis compared to PBS or NIH-3T3 groups (**Figure 1G**), indicating that CAFs could facilitate renal cancer proliferation and metastasis. Together, those results suggested that CAFs promoted tumor progression in renal cancer.

### CAFs Secreted Kyn to Regulate Renal Progression

**Figure 2:**
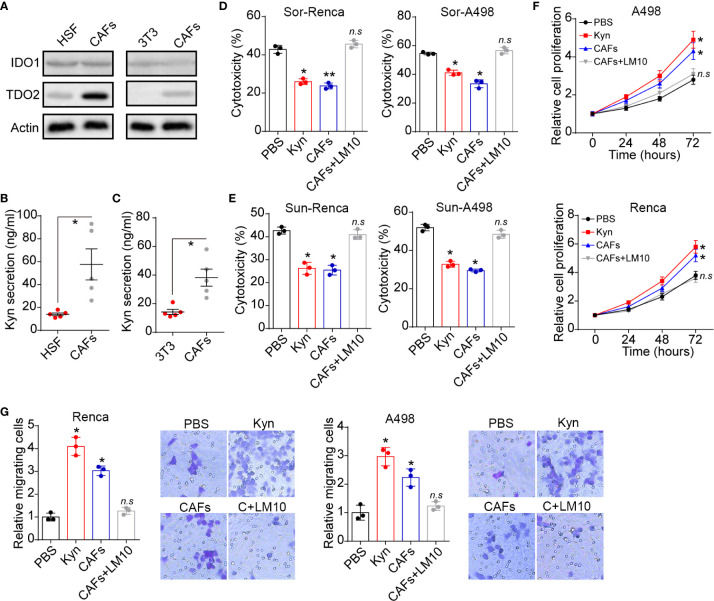
CAFs secreted Kyn to regulate tumor progression. **(A)** The western blotting of IDO1, TDO2 and β-actin in HSF, NIH-3T3 and CAFs isolated from renal patients (compared to HSF) or Renca bearing mice (compared to Renca). **(B)** The Kyn concentration in culture medium of HSF or CAFs isolated from patients using Elisa analysis (10^5^ cells in 2 ml culture medium, 48 hours). **(C)** The Kyn concentration in culture medium of NIH-3T3 or CAFs isolated from Renca bearing mice using Elisa analysis (10^5^ cells in 2 ml culture medium, 48 hours). **(D)** The cytotoxicity of Sor to Renca/A498 treated with Kyn (0.5 μM), CAFs or CAFs combined with LM10 (2 μM). **(E)** The cytotoxicity of Sun to Renca/A498 treated with Kyn (0.5 μM), CAFs or CAFs combined with LM10 (2 μM). **(F)** The relative cell proliferation of Renca/A498 treated with Kyn (0.5 μM), CAFs or CAFs combined with LM10 (2 μM). **(G)** The relative migrating cells numbers and representative images of Renca/A498 treated with Kyn (0.5 μM), CAFs or CAFs combined with LM10 (2 μM). Mean ± SEM, n.s, no significant difference; *p < 0.05, **p < 0.01.

Next, we further investigated the underlying mechanism of CAFs associated tumor progression. Compelling reports have provided evidence that tryptophan metabolism is tightly correlated to cancer development in diverse tumor types (15). Herein, we examined the expression of tryptophan metabolism associated dioxygenases indoleamine 2,3-dioxygenase 1 (IDO1) and TDO2. Notably, elevated expression of TDO2 was observed in CAFs compared to normal fibroblasts (**Figure 2A**). Consistently, increasing concentration of the tryptophan metabolite Kyn was found in CAFs culture medium (**Figures 2B, C**) compared to HSF or NIH-3T3 cells, indicating enhanced tryptophan metabolism in CAFs. To explore the effects of tryptophan metabolism to tumor cells, we added Kyn into the culture medium of cancer cells and found that Kyn efficiently promoted Sor resistance in Renca and A498 cells. Meanwhile, blockade of TDO by TDO inhibitor LM10 also suppressed the Sor resistance induced by CAFs (**Figure 2D**). The same phenomenon was observed in Sun treatment (**Figure 2E**), indicating that CAFs secreted Kyn to mediate multidrug resistance in renal cancer. Subsequently, we further examined the cells proliferation and migration of Renca/A498 cells by MTT and transwell analysis. Kyn treatment could significantly promote Renca/A498 cells proliferation and migration, whereas addition of LM10 retarded cells proliferation (**Figure 2F**) and migration (**Figure 2G**). Those results reminded that CAFs regulates renal cancer progression through Kyn.

### Kyn Produced by CAFs Promoted Cells Proliferation Through AhR/AKT Signaling

**Figure 3:**
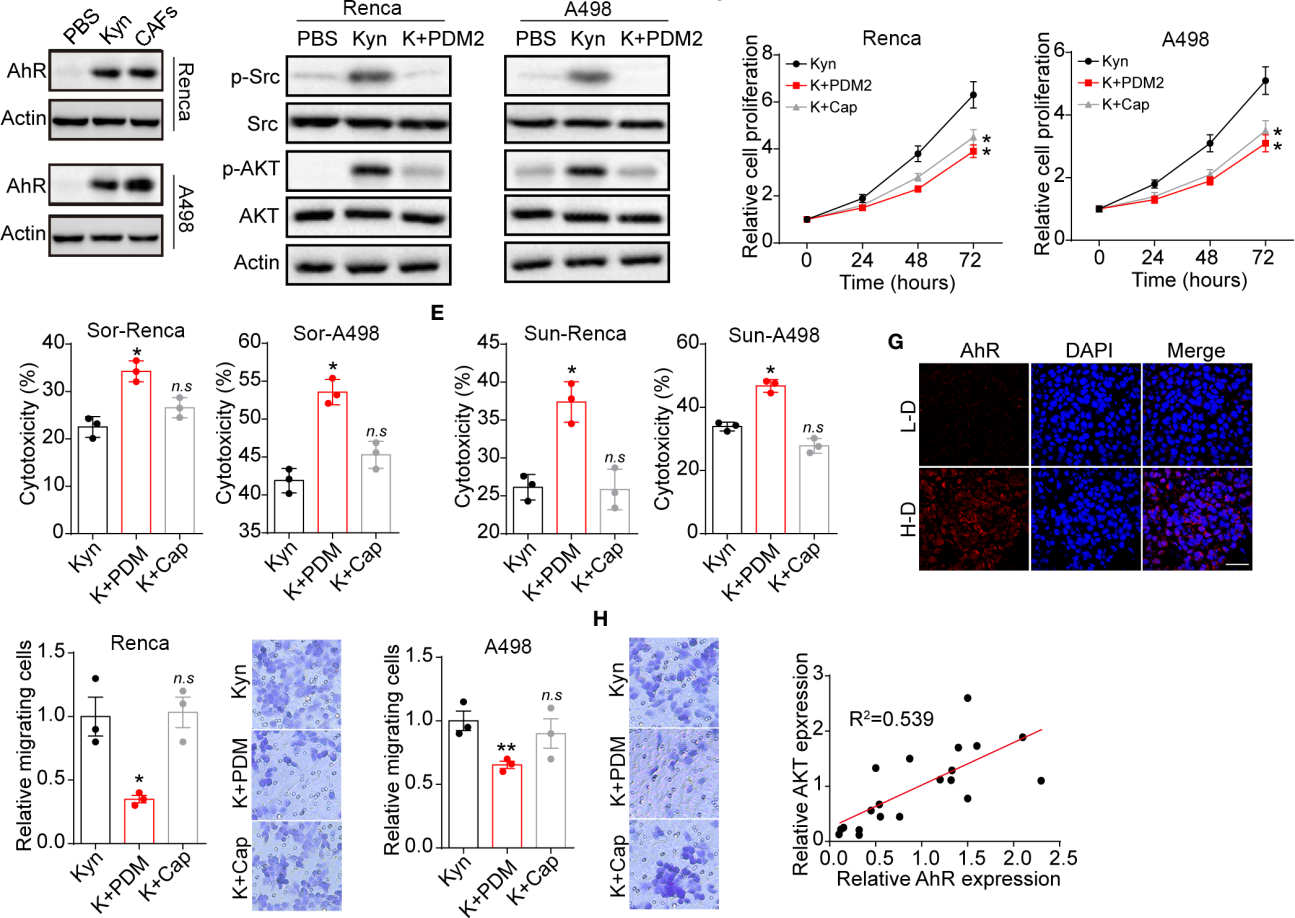
Kyn facilitated cells proliferation by AhR/ATK signal. **(A)** The western blotting of AhR and β-actin in Renca/A498 treated with PBS, Kyn (0.5 μM) or CAFs co-culture. **(B)** The western blotting of phosphorylated Src, total Src, phosphorylated AKT, total AKT and β-actin in Renca/A498 treated with PBS, Kyn (0.5 μM) or Kyn (0.5 μM) combined with PDM2 (1 nM). **(C)** The relative cells proliferation of Renca/A498 treated with Kyn (0.5 μM) or Kyn (0.5 μM) combined with PDM2 (1 nM)/Cap (10 nM). **(D)** The cytotoxicity of Sor to Renca/A498 treated with Kyn (0.5 μM) or Kyn (0.5 μM) combined with PDM2 (1 nM)/Cap (10 nM). **(E)** The cytotoxicity of Sun to Renca/A498 treated with Kyn (0.5 μM) or Kyn (0.5 μM) combined with PDM2 (1 nM)/Cap (10 nM). **(F)** The relative migrating cells numbers and representative images of Renca/A498 treated with Kyn (0.5 μM) or Kyn (0.5 μM) combined with PDM2 (1 nM)/Cap (10 nM). **(G)** Immunofluorescence staining of AhR in tumor tissues from high degree (H-D) and low degree (L-D) malignant renal patients. The scale bar is 50 μm. **(H)** The correlation analysis of AhR and phosphorylated AKT in tumor tissues from renal patients. Mean ± SEM, n.s, no significant difference, *p < 0.05, **p < 0.01.

To investigate the molecular mechanism of Kyn induced tumor progression, we further examined the Kyn downstream signal molecular in renal cancer cells. Intriguingly, elevated expression of AhR was observed in Kyn treated or CAFs co-cultured Renca/A498 cells (**Figure 3A**). More importantly, our immunofluorescent staining results indicated that Kyn treatment facilitated the nucleus entry of AhR in A498 cells (**Figure S1A**), indicating that Kyn induced AhR activation in renal cancer cells. Current study has suggested that activated AhR could mediate the crosstalk between JAK2 and Src (16, 17), resulting in the Src associated pro-survival signaling pathway activation. Herein, we further examined the expression of Src and Src downstream ATK signal. As a result, addition of Kyn efficiently mediated the activation of Src and AKT, whereas blockade of AhR by AhR inhibitor PDM2 reversed the phenomenon (**Figure 3B**). These results suggested that Kyn induced AKT signal activation through AhR axis. To further confirm the role of AhR/AKT axis in tumor progression, we applied AhR inhibitor PDM2 and AKT inhibitor Cap to treat Renca/A498 cells. Blockade of AhR or AKT obviously suppressed the cells proliferation induced by Kyn (**Figure 3C**), indicating that Kyn promoted cells proliferation through AhR/AKT signaling pathway. However, AKT inhibitor Cap treatment was not capable of suppressing the drugs resistance or cells migration (**Figures 3D–F**), reminding us that Kyn might regulate drugs resistance and migration through different AhR downstream signaling molecular. Next, we further confirmed the curial role of AhR in patients’ tumor tissues. The immunofluorescence staining implicated enhanced expression of AhR was observed in high degree malignant tumor tissues compared to low degree malignant tissues (**Figure 3G**). However, no significant difference of AKT expression was observed in H-D and L-D groups (**Figure S1B**), indicating that AKT associated signals might not be involved into the AhR associated drugs resistance in renal cancer. However, a potential correlation (R^2^>0.5) between AhR and AKT expression was still observed in those renal tumor tissues (**Figure 3H**), indicating that activation of AKT signals was AhR dependent in patients. Those results suggested that Kyn produced by CAFs promoted the cells proliferation through an AhR/AKT signaling pathway.

### Kyn Produced by CAFs Promoted Drugs Resistance and Cells Migration Through AhR/STAT3 Signaling

**Figure 4:**
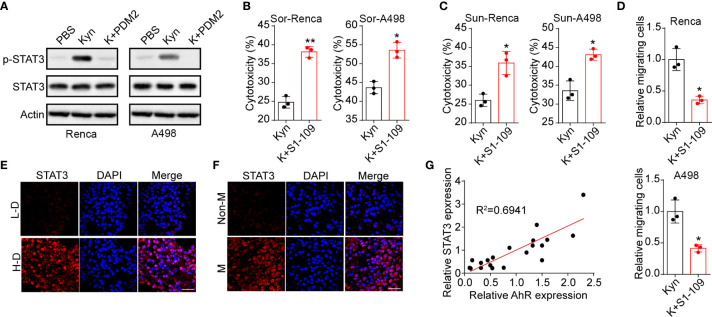
Kyn regulated cells migration and metastasis through AhR/STAT3 signal. **(A)** The western blotting of phosphorylated STAT3, total STAT3 in Renca/A498 treated with PBS, Kyn (0.5 μM), Kyn (0.5 μM) combined with PDM2 (1 nM). **(B)** The cytotoxicity of Sor to Renca/A498 treated with Kyn (0.5 μM) or Kyn (0.5 μM) combined with S1-109 (2 μM). **(C)** The cytotoxicity of Sun to Renca/A498 treated with Kyn (0.5 μM) or Kyn (0.5 μM) combined with S1-109 (2 μM). **(D)** The relative migrating cells numbers and representative images of Renca/A498 treated with Kyn (0.5 μM) or Kyn (0.5 μM) combined with S1-109 (2 μM). **(E)**, Immunofluorescence staining of phosphorylated STAT3 in tumor tissues from high degree (H-D) and low degree (L-D) renal cancer patients. The scale bar is 50 μm. **(F)** Immunofluorescence staining of phosphorylated STAT3 in tumor tissues from non-metastatic (non-M) and metastatic renal cancer patients. The scale bar is 50 μm. **(G)** The correlative analysis of AhR and phosphorylated STAT3 expression in tumor tissues from renal patients. Mean ± SEM, n.s, no significant difference; *p < 0.05, **p < 0.01.

STAT3, which serves as the Src downstream signaling molecular (18), is a crucial participant in tumor drugs resistance and distant metastasis (19). Herein, we examined the expression of STAT3 in Renca and A498 cells. Activation of STAT3 signal was observed in Kyn treated A498/Renca cells, whereas blockade of AhR suppressed the STAT3 activation (**Figure 4A**). More importantly, blockade of STAT3 signal by STAT3 inhibitor S1-109 obviously suppressed the drugs resistance (**Figures 4B, C**) and cells migration (**Figure 4D**) induced by Kyn. Additionally, we examined the cytotoxicity of our inhibitors, including LM10, PDM2, Cap and S1-109, to exclude the potential influence caused by inhibitors associated cytotoxicity. As a result, no significant cytotoxicity to tumor cells was observed in our inhibitors (**Figure S1C**). Next, the enhanced expression of phosphorylated STAT3 was also found in high degree malignant tumor tissues from renal cancer patients (**Figure 4E**) and tumor tissues from metastatic renal patients (**Figure 4F**), indicating that STAT3 signals are vital for tumor progression and distant metastasis in clinical renal cancer. The correlation analysis also implicated that the expression of phosphorylated STAT3 was related to the AhR expression (R^2^ = 0.6941) in clinical renal tumor tissues (**Figure 4G**). Together, those results suggested that Kyn regulated renal cancer drugs resistance and metastasis through AhR downstream STAT3 signaling pathway.

### Blockade of AhR Signals Improved Outcome of Chemotherapy in Mice Model

**Figure 5:**
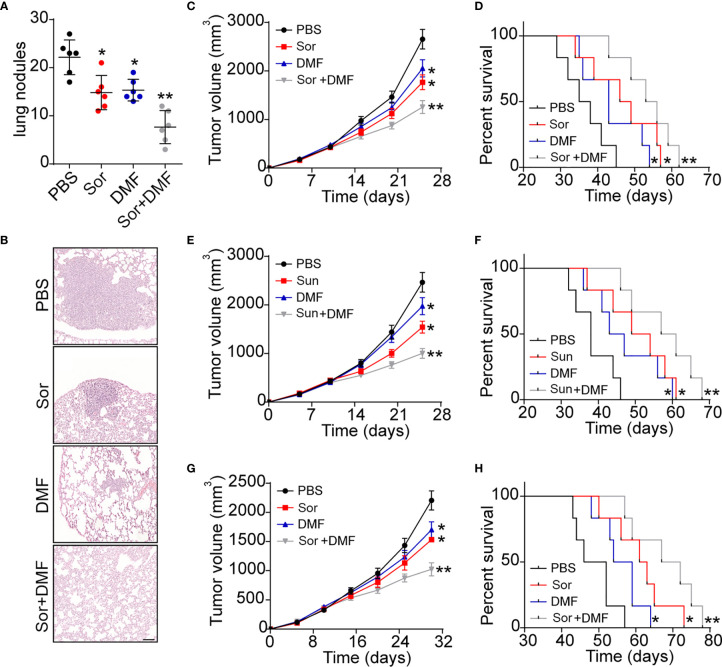
Blockade of AhR signals improved outcome of chemotherapy in renal cancer. **(A)** The metastasis lung nodules of Renca lung metastasis mice models treated with PBS, DMF, Sor and Sor combined with DMF. **(B)** Representative images of lung tissues from **(A)**. The scale bar is 200 μm. **(C)** The tumor volume of subcutaneous Renca bearing mice treated with PBS, DMF, Sor and Sor combined with DMF. **(D)** The survival time of subcutaneous Renca bearing mice treated with PBS, DMF, Sor and Sor combined with DMF. **(E)** The tumor volume of subcutaneous Renca bearing mice treated with PBS, DMF, Sun and Sun combined with DMF. **(F)** The survival time of subcutaneous Renca bearing mice treated with PBS, DMF, Sun and Sun combined with DMF. **(G)** The tumor volume of subcutaneous A498 bearing mice treated with PBS, DMF, Sor and Sor combined with DMF. **(H)** The survival time of subcutaneous A498 bearing mice treated with PBS, DMF, Sor and Sor combined with DMF. Mean ± SEM, n.s, no significant difference; *p < 0.05, **p < 0.01.

Given the crucial role of AhR in renal cancer progression, it might be feasible to block AhR signals to improve the outcome of Sor/Sun therapy. DMF, an AhR inhibitor, revealed obvious anti-cancer activity and could be administered orally, which is befitting for renal cancer therapy. Herein, we established lung metastatic/subcutaneous Renca bearing mice model and treated mice with PBS, DMF and Sor. As anticipated, blockade of AhR signals by DMF efficiently suppressed the lung metastasis of Renca cells. Meanwhile, DMF/Sor combination group revealed enhanced anticancer effects compared to the single DMF or Sor groups (**Figures 5A, B** and **S1D**). Addition of DMF significantly strengthened the tumor suppressive effects of Sor and prolonged the survival time in subcutaneous Renca bearing mice (**Figures 5C, D**). The synergistic effects were also observed in DMF and Sun combination (**Figures 5E, F**). Next, we further established human derived renal cancer model by subcutaneously injecting A498 into immunodeficient mice. Similarly, combination of DMF and Sor significantly suppressed the A498 tumor growth and prolonged the overall survival time of mice (**Figures 5G, H**). Taken together, those results suggested that blockade of DMF could strengthen the anticancer effects of chemotherapy, which provided an innovative approach for clinical renal cancer treatment.

## Discussion

Despite the advance in cancer diagnose and therapy, the role of CAFs in renal cancer development remains controversial. CAFs paly a complex role in cancer development through secretion of diverse elements (20). Previous reports focused on the cytokines derived from CAFs, which influenced tumor cells through activation of pro-survival signaling pathway directly. However, cellular metabolism is emerging as critical participant in tumor biological activities, which is frequently associated with tumor progression (21). Here, our study identified the role of CAFs in renal cancer progression, which was dependent on the Kyn associated signaling pathway.

Our study proved that CAFs isolated from tumor tissues contributed to the renal cancer drugs resistance and tumor progression, whereas normal fibroblasts failed to facilitate tumor development *in vitro* and *in vivo*. The Elisa and western blotting analysis suggested that CAFs isolated from tumor tissues revealed strengthened capability of tryptophan metabolism, contributing to the elevated secretion of Kyn in CAFs compared to normal fibroblasts. The Kyn derived from CAFs could efficiently mediate the activation of pro-survival signaling pathways in renal cancer, eventually resulting in cancer development. Compelling reports focused on the cytokines produced by CAFs, such as IGF2, EGF, IL-6 and so on (14). Wang and his colleagues reported that CAFs facilitated cells metastasis of lung cancer through the IL-6/JAK/STAT signaling pathway (22). Apart from cytokines, proteins or compounds produced by CAFs might participate in the tumor process regulation. Li reported that CAFs could facilitate drugs resistance of breast cancer through secretion of type I collagen (23). However, the influence of fibroblasts metabolism to tumor progression has yet to be explored. Kyn has been demonstrated to be an immunosuppressive regulator in innate and adaptive immune response, which is highly correlated to the immune tolerance in cancer development (24). Our study further provided evidence to described the role of Kyn in CAFs associated renal cancer progression. We proved that Kyn produced by CAFs could promote the AhR activation in tumor cells and contribute to the downstream pro-survival signals activation in renal cancer.

**Figure 6:**
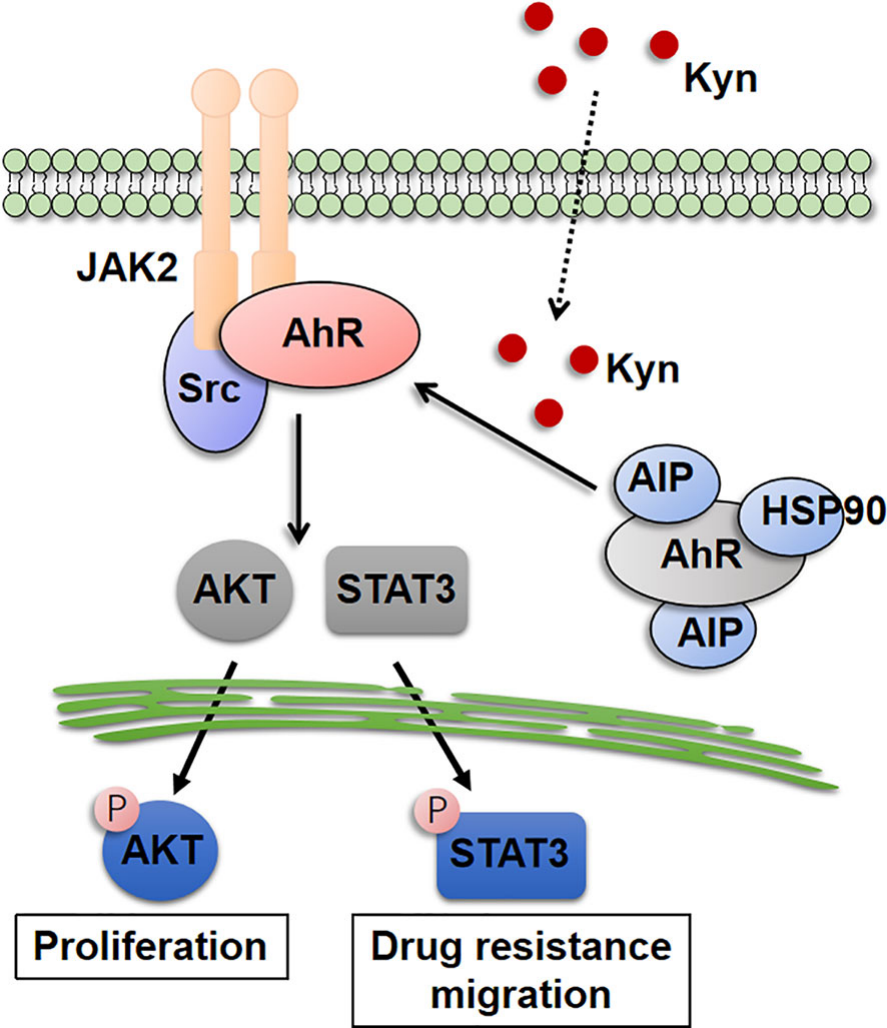
The schematic diatgram of CAFs induced renal cancer progression.

AhR, recognized as a ligand-activated basic helixloop-helix transcriptional factor that responses to environmental alternations, has been reported to be associated with tumor growth, drugs resistance and tumor immunosuppression (25). GJ Prud’homme reported that the expression of AhR referred to the cancer stem cells in breast cancer (26). Meanwhile, the sustained activation of the AhR transcription factor could mediate the resistance to BRAF-inhibitors in melanoma (27). Clinical data also suggested that the expression of AhR is highly correlated to the EGFR-TIKs resistance in non-small cell lung cancer (17). Simultaneously, several reports implicated that inhibition of AhR signaling by targeting the AhR proteins or associated ligands is prone to suppress tumor growth and recurrence. In our study, we proved that Kyn derived from CAFs could mediate the AhR activation in renal cancer, contributing to the downstream AKT and STAT3 signaling pathway activation (**Figure 6**). Our study further used AhR inhibitor DMF to be combined with Sor/Sun therapy, which revealed improved tumor suppressive effects and metastasis inhibition. Compared to previous AhR inhibitors, DMF revealed enhanced tumor suppressive effects, as well as good safety. No significant weight loss was observed in our DMF treated mice. Meanwhile, oral administration of DMF enabled the clinical application in renal therapy, which is more appreciate to be combined with Sor/Sun (oral administration) and provides potential clinical implication.

In conclusion, our study demonstrated that CAFs produced Kyn to promote tumor progression and drugs resistance in renal cancer, which is dependent on the AhR/AKT/STAT3 signaling pathway. Blockade of AhR by DMF could significantly improve the anticancer effects of Sor/Sun, which described a novel strategy for clinical renal cancer therapy.

## Data Availability Statement

The original contributions presented in the study are included in the article/supplementary material. Further inquiries can be directed to the corresponding author.

## Ethics Statement

The studies involving human participants were reviewed and approved by the committee of the First Affiliated Hospital, University of South China. The patients/participants provided their written informed consent to participate in this study. The animal study was reviewed and approved by the Institute Ethics Committee of the First Affiliated Hospital, University of South China.

## Author Contributions

RH conceived the project and wrote the manuscript. L-bC, S-pZ, T-pL, HZ, and Y-jD performed the experiments. L-bC, P-fC, and Y-jD performed data analysis. All authors contributed to the article and approved the submitted version.

## Funding

This study was supported by Scientific Research Fund Project of Hunan Provincial Health Commission 20200540.

## Conflict of Interest

The authors declare that the research was conducted in the absence of any commercial or financial relationships that could be construed as a potential conflict of interest.
