# Supplementary material for: Cancer Associated Fibroblasts Promote Renal Cancer Progression Through a TDO/Kyn/AhR Dependent Signaling Pathway
Source: Front Oncol. 2021 Mar 25;11:628821. doi: 10.3389/fonc.2021.628821 (PMC8027476; doi:10.3389/fonc.2021.628821)
Supplement: Supplementary file 1 [file DataSheet_1.docx]

**Supplementary information**

Supplementary figure 1


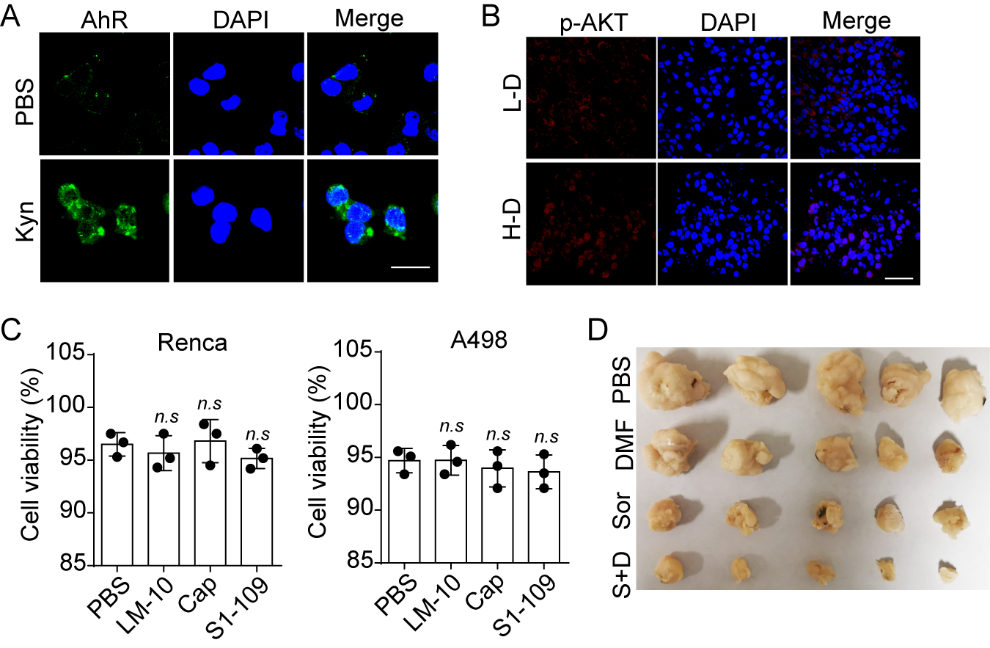


Supplementary figure 1 A, immunofluorescence staining of AhR in A498 cells treated with PBS or Kyn (0.5 μM). The scale bar is 15 μm. B, immunofluorescence staining of phosphorylated AKT in tumor tissues from high degree (H-D) and low degree (L-D) renal cancer patients. The scale bar is 50 μm. C, cells viability of Renca and A498 cells treated with PBS, LM10 (2 μM), PDM2 (1 nM), Cap (10 nM) or S1-109 (2 μM). D, the tumor images of subcutaneous Renca bearing mice treated with PBS, DMF, Sor and Sor combined with DMF. n.s means no significant difference.
